# Convergent alteration of lung tissue microbiota and tumor cells in lung cancer

**DOI:** 10.1016/j.isci.2021.103638

**Published:** 2021-12-16

**Authors:** Hui Dong, Qiang Tan, Yuanyuan Xu, Yongqiang Zhu, Yaxian Yao, Yuezhu Wang, Chong Li, Hong Li, Guoqing Zhang, Yan Xiong, Meihua Ruan, Jiadong Zhao, Weirong Jin, Lungen Lu, Shun Lu

**Affiliations:** 1 Department of Gastroenterology, Shanghai Key Laboratory of Pancreatic Diseases, Shanghai General Hospital, Shanghai Jiao Tong University School of Medicine, No. 650 Xin Songjiang Road, Shanghai 201620, China; 2 Department of Shanghai Lung Cancer Center, Shanghai Chest Hospital, Shanghai Jiao Tong University, No. 241 West Huaihai Road, Shanghai 200030, China; 3 Chinese National Human Genome Center at Shanghai, Shanghai 201203, China; 4 Cancer Institute, Fudan University Shanghai Cancer Center, Shanghai 200032, China; 5 Bio-Med Big Data Center, Key Laboratory of Computational Biology, CAS-MPG Partner Institute for Computational Biology, Shanghai Information Center for Life Sciences, Shanghai Institute of Nutrition and Health, Shanghai Institutes for Biological Sciences, University of Chinese Academy of Sciences, Chinese Academy of Sciences, Shanghai 200031, China; 6 Nanjing Shenyou Institute of Genome Research, Nanjing 210048, China

**Keywords:** Health sciences, Microbiome, Cancer

## Abstract

Microbiota-host interaction plays an important role in cancer predisposing, initiation, progression, and response to therapy. Here, we explored the composition of lung tissue microbiota in 143 Chinese patients through conducting 16S rRNA gene sequencing, while TP53 mutation in tumor cells was assessed simultaneously. We found PAH-degrading microbes were more abundant in lung tumor microbiota from smokers. Furthermore, TP53 mutation was more prevalent in smokers, and TP53-mutated tumor harbored more *Massilia*, as well as *Acidovorax* that was also capable of degrading PAH. Further analysis showed DNA recombination and repair pathway was enriched in microbiota of smokers, which was convergent to the alteration occurred in tumor cells. Meanwhile, the microbiota of TP53-mutated tumor also exhibited dysregulation of p53 signaling pathway. Our results provided insights into the association of lung commensal microbes with tobacco exposure and host gene mutation, suggesting microbiota and tumor cells might undergo convergent alteration and mutually benefit each other.

## Highlights

• PAH-degrading microbes were more abundant in lung tumor microbiota from smokers
• p53 signaling pathway was dysregulated in microbiota from TP53-mutated lung tumors
• Microbiota and host cell in tumor microenvironment underwent convergent alteration

## Graphical abstract

**Figure:**
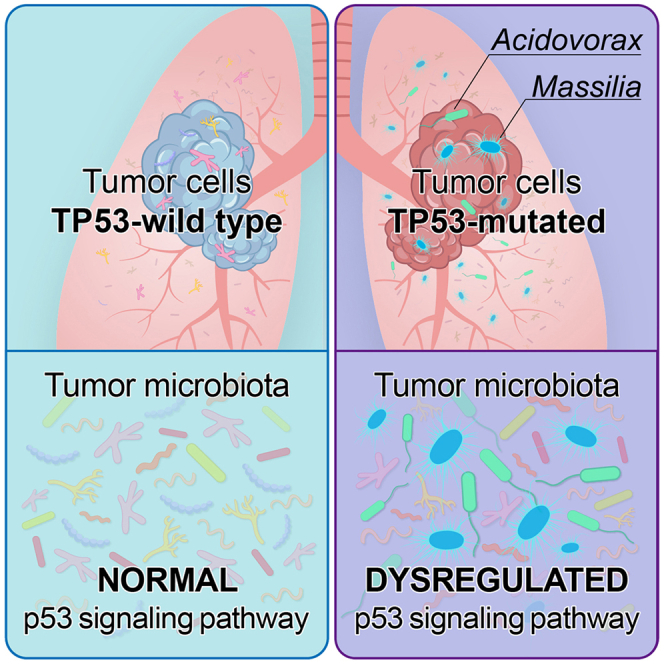

## Introduction

Our body harbors abundant and diverse microbiota on all the surface barriers, including skin, nasal cavity, oral cavity, pharynx, gut, and vagina. It was suggested that microbiota might be involved in cancer at multiple levels including predisposing conditions, initiation, progression, susceptibility to host immune response, and response to therapy (Dzutsev et al., 2017). Lung was thought to be sterile as no culturable bacteria were available from lower respiratory tract samples (Baughman et al., 1987; Thorpe et al., 1987). However, with the development of culture-independent techniques, especially the widespread application of next-generation sequencing, it was uncovered that lung is also the home to diverse communities of microbes (Dickson and Huffnagle,2015). Microbiota may play an important role in maintaining lung homeostasis. It was demonstrated that microbiota in upper respiratory tract of mice could inhibit excessive lung immune response to acute influenza infection through promoting the differentiation of M2 macrophages (Wang et al., 2013). Although dysbiosis of lung microbiota has been linked to lung cancer, their roles in tumorigenesis and/or progression have not been revealed comprehensively (Wong-Rolle et al., 2021). Recently, Jin et al. reported that lung microbiota could promote inflammatory responses and tumor cell proliferation by activating lung-resident γδ T cells in mouse models, which established a clearly association between local microbiota-immune crosstalk and cancer development (Jin et al., 2019).

Up to now, the majority of studies on human lung microbiota were carried out using samples of sputum, bronchoscopic brushing, or bronchoalveolar lavage (BAL) which could not avoid potential contamination by the upper airway microbiota (Berger and Wunderink, 2013). It was indicated that taxonomic composition of lung microbiota could be entirely discordant between tissue-based samples and BAL samples (Willner et al., 2012; Bingula et al., 2020; Reinhold et al., 2020). Thus, studies involving tissue samples would be of importance when investigating lung microbiota. In recent years, studies on human lung cancer tissue samples have been carried out and showed that composition of lung microbe community was distinct from that of other body sites such as oral, nasal, gut, skin, and vagina (Yu et al., 2016). Besides, it also differed from other cancer types including breast, ovary, pancreas, melanoma, bone, and brain tumors (Nejman et al., 2020). Interaction between microbiome and host gene was investigated, revealing unique characteristics of microbiota in tumors harboring TP53 mutations (Greathouse et al., 2018). However, tissue samples included in previous studies were obtained from USA, Italy, Israel, and Netherlands, and the characteristics of microbiota of cancerous lung tissue in Chinese remained undiscovered. Besides, more evidences were also needed to further reveal the relationship of microbes and host cells in lung cancer.

In this study, we characterized features of lung tissue microbiota through 16S rRNA gene sequencing of 241 samples from 143 Chinese patients, including 118 tumor and 123 non-tumor lung tissue samples. We assessed the association between lung tissue microbiota and smoking, and the correlation of TP53 mutations in tumor cells and commensal microbes was also investigated. Our results suggested microbiota in tumor microenvironment and host cells might undergo convergent alteration and mutually benefit each other.

## Results

### 16S rRNA and TP53 gene sequencing results of lung tissue samples

**Figure 1:**
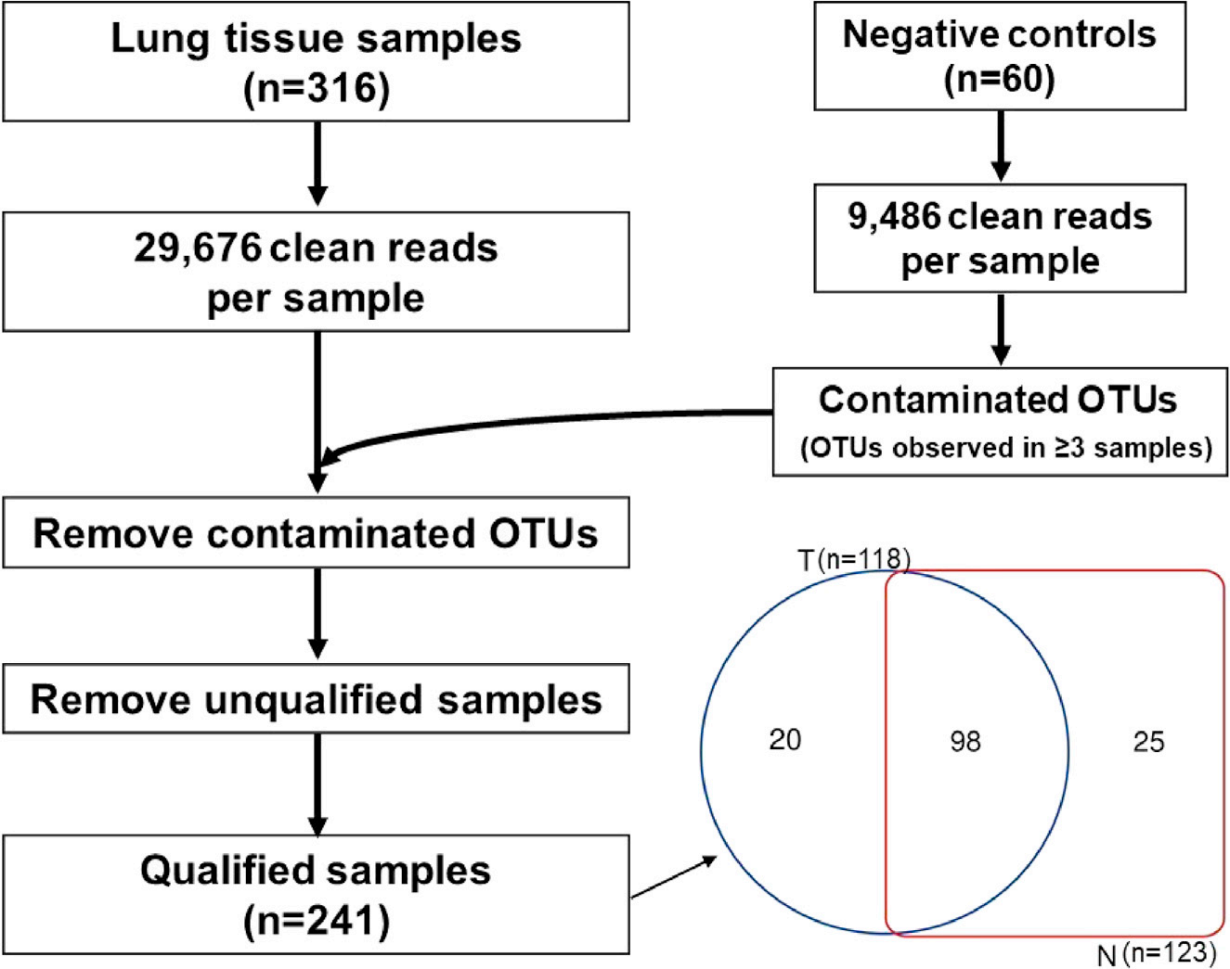
Flowchart of qualifying 16S rRNA sequencing results A total of 158 pairs of tumor/non-tumor lung tissue samples (n = 316) were subjected to 16S rRNA gene sequencing. Sixty negative controls were included simultaneously in each experimental procedure such as DNA extraction, 16S rRNA amplification, and sequencing. OTUs presented in ≥3 negative controls were regarded as contamination and removed from sequencing results of lung tissue samples. A total of 241 lung tissue samples from 143 patients were qualified for further analysis which included 98 pairs of tumor/non-tumor samples, 20 tumor samples, and 25 non-tumor samples. N, non-tumor tissue; T, tumor tissue. See also Tables S2 and S3.

A total of 316 lung tissue samples (*i.e.* 158 pairs of tumor/non-tumor samples) were collected from 158 patients who underwent thoracoscopic lobectomies and subjected to 16S rRNA gene sequencing. The average number of sequencing reads per sample was 29,676 ± 10,537 (mean ± standard deviation). For 60 negative control samples, an average of 9,486 ± 25,020 clean reads per sample was obtained. After removing possible contaminated OTUs as described in “STAR Methods”, a total of 241 lung tissue samples from 143 patients were qualified for further analysis which included 98 pairs of tumor/non-tumor samples, 20 tumor samples, and 25 non-tumor samples (Figure 1).

Clinical characteristics of 143 patients were described in Tables 1 and S1. Briefly, among the 143 subjects, 51.8% (*n* = 74) were male and 48.2% (*n* = 69) were female, with a mean age of 60.6 ± 10.7 years. A total of 42 (29.4%) individuals were smokers and 101 (70.6%) were non-smokers. The histological subtype of most patients (107 out of 143, 74.8%) was adenocarcinoma (AD), while only 22 (15.4%) was squamous cell carcinoma (SCC). The other 14 (9.8%) patients had miscellaneous lung tumors other than AD and SCC. TP53 gene was also sequenced in both tumor and non-tumor tissues, with an average sequencing depth >500X. There were 43 (30.1%) and 100 (69.9%) patients who were positive and negative for TP53 somatic mutation, respectively.

**Table 1:** Characteristics of 143 patients

Histopathologic subtype	No. of patients	Mean age±SD	Gender (male/female)	Smoking status (smoker/non-smoker)	TP53 somatic mutations (positive/negative)
Adenocarcinoma	107	59.7±10.1	40/67	17/90	25/82
SCC^a^	22	66.1±8.2	22/0	14/8	13/9
Miscellaneous	14	59.3±15.9	12/2	11/3	5/9
Subtotal	143	**60.6±10.7**	**74/69**	**42/101**	**43/100**

See also Table S1.

a SCC, squamous cell carcinoma.

### Taxonomic composition of lung tissue microbiota

**Figure 2:**
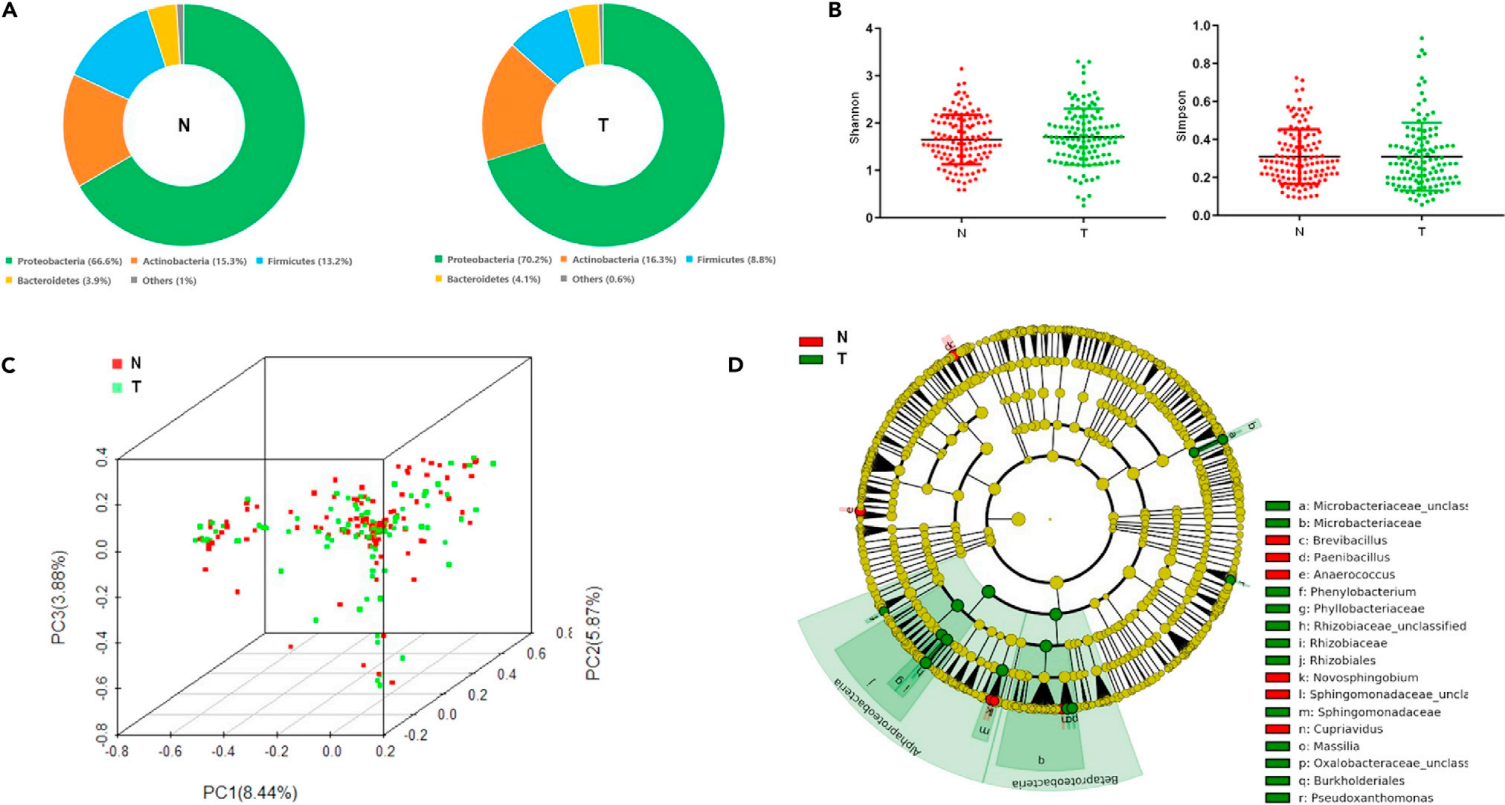
Comparison of lung microbiota between non-tumor and tumor tissues (A) The microbiota of lung tissue in Chinese patients was composed of *Proteobacteria*, *Actinobacteria*, *Firmicutes*, and *Bacteroidetes*, with *Proteobacteria* being the most abundant taxon in both non-tumor (N) and tumor (T) tissues. (B) No significant difference was observed in Shannon (left) and Simpson (right) index between non-tumor (N) and tumor (T) lung tissues. (C) Clustering of microbiota between non-tumor (N) and tumor (T) lung tissues by principal coordinate analysis. (D) Differentially abundant microbial taxa between non-tumor (N) and tumor (T) lung tissues. *Massilia* was more abundant in tumor compared with non-tumor tissues. See also FiguresS1 and S2.

The microbiota composition was then analyzed in 241 qualified lung tissue samples which included 118 tumor and 123 non-tumor samples. Consistent with previous studies (Reinhold et al., 2020; Nejman et al., 2020; Sze et al., 2015; Kim et al., 2017), the most dominant taxa in lung tissue microbiota were *Proteobacteria*, *Actinobacteria*, *Firmicutes*, and *Bacteroidetes* at phylum level (Figure 2A). There was no significant difference in proportion of these taxa between tumor and non-tumor samples (Figure S1A). Alpha diversity indicated by Shannon and Simpson index did not show significant difference between these two groups (Figure 2B). However, significant difference was observed in beta diversity between tumor and non-tumor samples (*p* = 0.001, Figures 2C and S1B). *Massilia*, *Phenylobacterium*, and *Pseudoxanthomonas* were more abundant in tumor tissue, while the abundance of *Brevibacillus*, *Cupriavidus*, and *Anaerococcus* were higher in non-tumor tissue samples (Figure 2D). We found *Brevundimonas*, *Ruminococcus*, and *Polaromonas* were among the differentially abundant bacterial taxa between AD and SCC (Figure S2), which were also included in the taxonomic consortia differentiating histological subtypes of lung cancer as previously reported (Greathouse et al., 2018).

### Characteristics of microbiota in tumor tissues of smokers

**Figure 3:**
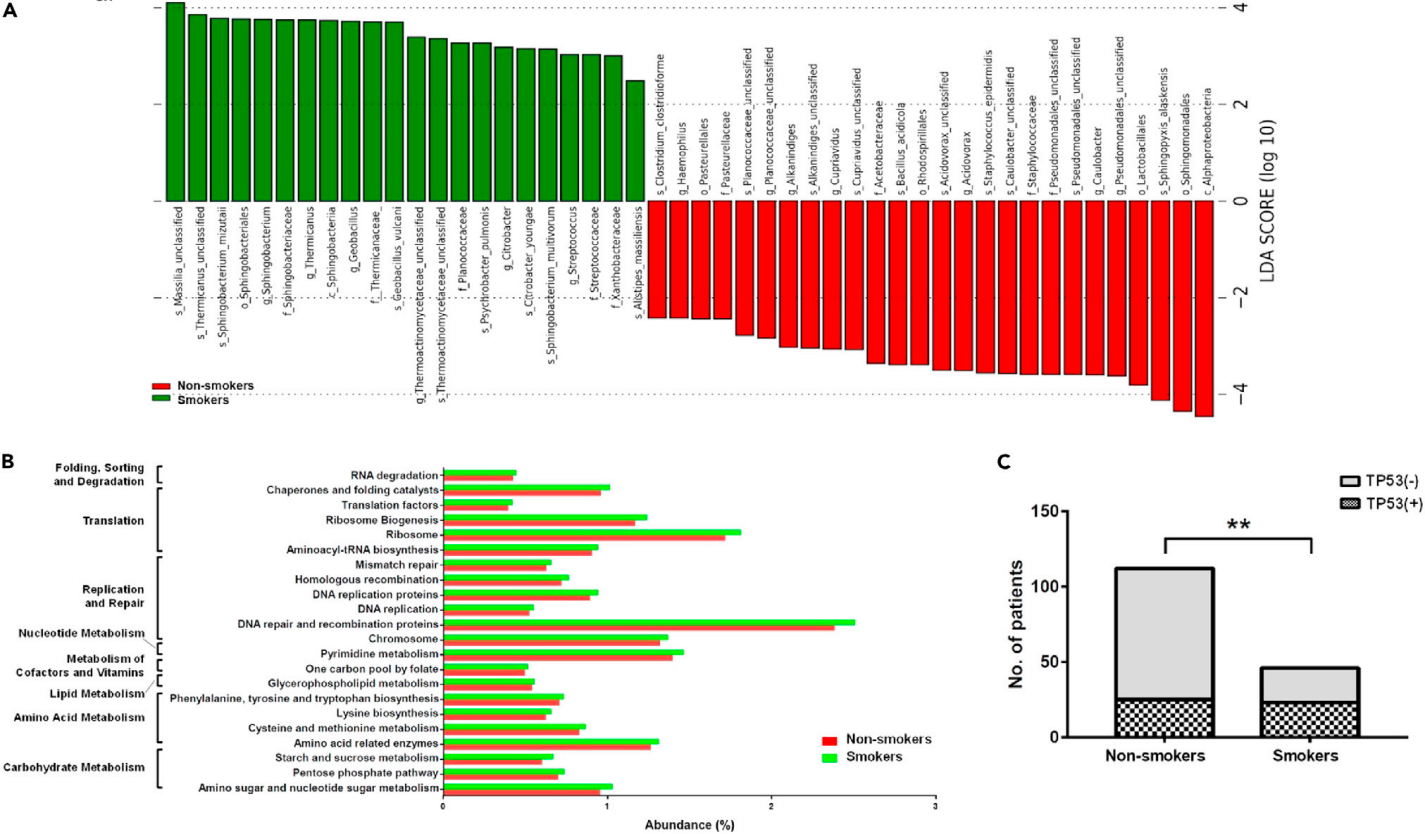
Differential composition of lung microbiota in tumor tissues of non-smokers and smokers (A) Differentially abundant microbial taxa between non-smokers and smokers in tumor tissues included *Massilia*, *Sphingobacterium*, and *Acidovorax* which were capable of degrading PAH. (B) Functional prediction based on PICRUSt analysis of 16S rRNA gene taxonomic data. The microbiota of tumor tissues was enriched in pathways related to DNA recombination and repair in smokers. (C) The prevalence of TP53 mutation was significantly higher in smokers than in non-smokers (∗∗ represents *p*<0.01, Chi-Square test).

Compared with non-smokers, tumor microbiota of smokers was more abundant in *Massilia* and *Sphingobacterium* while less abundant in *Acidovorax* (Figure 3A). Notably, *Massilia*, *Sphingobacterium*, and *Acidovorax* are capable of degrading polycyclic aromatic hydrocarbon (PAH) which is a well-known carcinogen in cigarette smoke. Functional prediction based on PICRUSt analysis of 16S rRNA gene taxonomic data indicated that tumor microbiota of smokers was enriched in pathways related to DNA recombination and repair, translation, and metabolism of carbohydrate, lipid, and amino acid (Figure 3B). As smoking would cause DNA damage to cells, and cells would respond by activating DNA repair to remove DNA lesions, enrichment of DNA recombination and repair pathway in commensal microbiota of smokers seemed to be convergent to the alteration occurred in host cells. As TP53 is the most commonly mutated gene in lung cancer (Robles and Harris, 2010), we examined TP53 somatic mutations and found that 50% of smokers (21 out of 42) and 22% (22 out of 101) of non-smokers were positive for TP53 mutation, indicating TP53 mutation was more prevalent in tobacco-related lung cancer (Figure 3C).

### Convergent alteration of lung tissue microbiota and tumor cells

As shown in Table 1, there were 13 out of 22 (59%) patients with SCC who were TP53 positive, while 23% (25 out of 107) and 36% (5 out of 14) of patients with AD and miscellaneous lung tumors were TP53 positive, respectively. The percentage of patients who were TP53 positive was significantly higher in SCC than in AD (p<0.01, Fisher's exact test). However, when stratifying patients by different pathological types, no microbial taxa differentially abundant between TP53 mutation-positive tumors (n = 13) and TP53 mutation-negative tumors (n = 9) was found in SCC due to limited number of patients, and it was the same in patients with miscellaneous lung tumors. Therefore, comparisons were carried out between TP53 mutation-positive tumors and TP53 mutation-negative tumors without pathological stratification.

**Figure 4:**
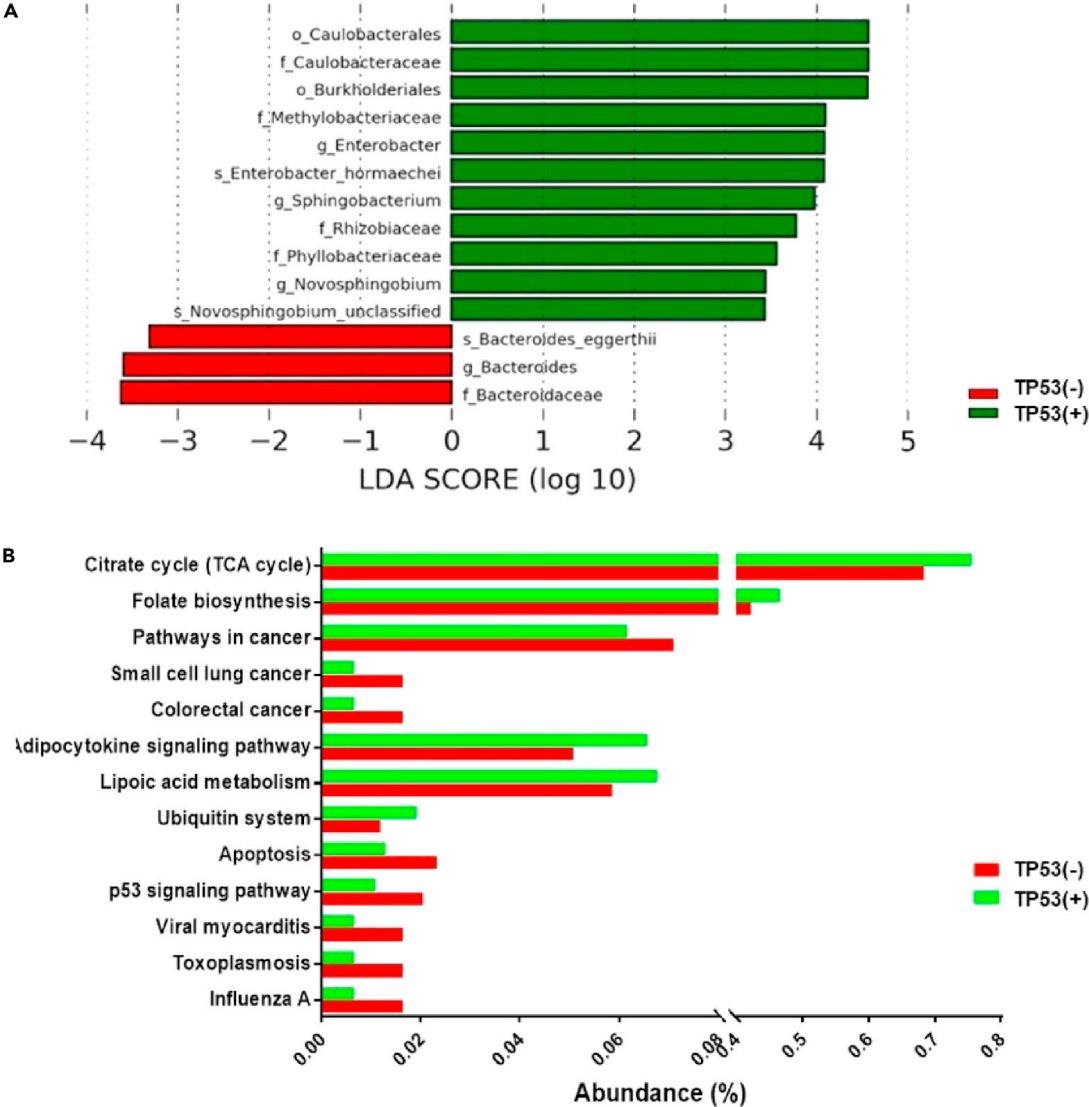
Microbial dysbiosis in TP53 mutation-positive tumor tissues (A) *Sphingobacterium* was more abundant in TP53 mutation-positive tumor tissues than in paired non-tumor tissues. (B) The microbiota of TP53 mutation-positive tumor tissues was less abundant in p53 signaling pathway and apoptosis pathway compared with paired non-tumor tissues. TP53 (+) represents TP53 mutation-positive tumor tissues and TP53 (−) represents paired non-tumor tissues which were TP53 mutation-negative. See also Figure S3.

It was demonstrated that tumors harboring TP53 mutations had a unique bacterial consortium featured by high abundance of *Acidovorax* (Greathouse et al., 2018). Consistently, we also observed *Acidovorax*, as well as *Massilia* were enriched in TP53 mutation-positive tumors compared with mutation-negative tumors (Figure S3). Compared with its paired non-tumor tissue, the abundance of *Sphingobacterium* was higher in tumor tissue with TP53 somatic mutations (Figure 4A). Functional prediction showed p53 signaling pathway and apoptosis pathway were downregulated in microbiota of TP53 mutation-positive tumor tissues (Figure 4B). Such functional alterations resembled that of tumor cells, which were also aberrant in p53 signaling and apoptosis due to TP53 mutations, reflecting a convergent alteration in commensal microbes and tumor cells.

## Discussion

Knowledge on lung tissue microbiota was still very limited up to now. Previous studies revealed the lung tissue microbiota was dominated by *Proteobacteria* in Koreans, Italians, Canadians, and Americans (Reinhold et al., 2020; Nejman et al., 2020; Sze et al., 2015; Kim et al., 2017). In the present study, we analyzed the characteristics of lung tissue microbiota in tumor and non-tumor tissues obtained from Chinese patients. We found microbiota of lung tissue in Chinese was composed of *Proteobacteria*, *Actinobacteria*, *Firmicutes*, and *Bacteroidetes*, with *Proteobacteria* being the most abundant taxon in both tumor and non-tumor tissues. Thus, domination of *Proteobacteria* in lung tissue microbiota seemed to be consistent regardless of different countries. To be noted, as lung tissue samples were regarded as that of low microbial biomass, special precautions were taken in this study to avoid potential contaminations, with experiments carrying out in several rooms physically separated and individually ventilated, and multiple controls being involved in experimental procedures of DNA extraction, 16S rRNA gene amplification, and sequencing. Through removing OTUs presented in ≥3 negative controls, we maximumly eliminated potential contaminated microbial taxa from the sequencing results of lung microbiota.

The genus *Massilia* and *Sphingobacterium* were not only widely distributed in environmental sources, but also has been isolated from human clinical specimens (Orthováet al., 2015; Lindquist et al., 2003; Huys et al., 2017). We observed *Massilia* was more abundant in tumor than in non-tumor tissues, suggesting tumorigenesis may provide a microenvironment conducive to the growth of *Massilia*. Additionally, both *Massilia* and *Sphingobacterium* were enriched in tumor tissues of smokers compared with that of non-smokers, indicating that smoking might create an environment that allowed them to outcompete other species for resources and survive. To be noted, either *Massilia* or *Sphingobacterium* could efficiently degrade phenanthrene, a kind of PAH that is a well-known carcinogen in cigarette smoke responsible for lung cancer (Lou et al., 2016; Boffetta et al., 1997; Parab and Phadke, 2020). Thus, our result was consistent with the recent study showing bacterial taxa of smokers were enriched in degradation pathways for chemicals in cigarette smoke (Nejman et al., 2020). We proposed that lung commensal microbiota might counteract the exposure of cigarette smoke through increasing the abundance of bacterial degrading PAH which would benefit host cells by protecting them from PAH injury.

*Acidovorax*, which was also able to degrade PAH, has been identified to be enriched in smokers in SCC specifically, but not in AD (Greathouse et al., 2018). However, our results indicated *Acidovorax* was more abundant in non-smokers. Such inconsistence might be derived from the fact that the majority of patients (74.8%) involved in our study were AD. Furthermore, *Acidovorax* did not differentiate smokers and non-smokers in a healthy population (Yu et al., 2016). These results suggested the distribution of *Acidovorax* in smokers and non-smokers varied in different histological subtypes. Nevertheless, Greathouse et al. further revealed *Acidovorax* exhibited higher abundance among the subset of SCC with TP53 mutations (Greathouse et al., 2018). Consistently, we found that compared with those without TP53 mutations, TP53 mutation-positive tumor tissues harbored more *Acidovorax* as well as *Massilia*. It was demonstrated that TP53 mutation in lung cancer was attributed to direct DNA damage from cigarette smoke carcinogens (Pfeifer et al., 2002), and our results showed TP53 mutations were more prevalent in tobacco-related lung cancer. Thus, it was likely that the enrichment of *Acidovorax* and *Massilia* in tissues with TP53 mutations was a consequence of cigarette smoke exposure. In fact, we did observe *Massilia* was enriched in response to cigarette smoke exposure as discussed above. We speculated that commensal microbes and host cells might mutually benefit each other upon cigarette smoke exposure. On one hand, as the composition of microbial community altered with increased abundance of PAH-degrading bacteria upon cigarette smoke exposure, it implied commensal microbiota might benefit host cells by eliminating PAH-induced DNA damage. On the other hand, PAH-induced TP53 mutations in host cells would impair its normal function as a tumor suppressor and allow evasion of cell death, while living cells are critical to the survival of commensal bacteria.

When DNA damage occurred upon cigarette smoke exposure, DNA repair process would be enhanced consequently in host cells (Roos et al., 2016). Interestingly, we found DNA recombination and repair pathway was enriched in tumor microbiota of smokers compared with non-smokers, implying that microbiota and host cells might undergo a convergent alteration in response to carcinogen exposure. Furthermore, we also found p53 signaling pathway and apoptosis pathway were downregulated in lung microbiota of TP53 mutation-positive tumor tissues compared with paired non-tumor tissues which was TP53 mutation-negative. As TP53 mutation-positive tumor cells were also aberrant in p53 signaling and apoptosis, this finding further indicated that microbiota in tumor microenvironment tended to be functionally assimilated to host cells. It was demonstrated that gene mutations in host cells could alter gastrointestinal microbiomes, while microbiota may switch functional activity of host gene mutants from tumor-suppressive to oncogenic, indicating an intimate interaction between microbes and host cells (Meeker et al., 2020; Kadosh et al., 2020) Our results suggested such interaction might finally result in a convergent status that microbiota in tumor microenvironment gained coherent functions with host cells. Nevertheless, more evidences are needed to prove this point of view.

In summary, we showed lung tissue microbiota was dominated by *Proteobacteria* in Chinese patients. The composition of lung commensal microbiota was affected by exposure to cigarette smoke with enrichment of PAH-degrading bacteria such as *Massilia* and *Sphingobacterium*. Compared with mutation-negative tumors, TP53 mutation-positive tumor tissues harbored more *Massilia* and *Acidovorax* which was also capable of degrading PAH. Further analysis showed lung microbiota in tumor microenvironment tended to be functionally assimilated to host cells. Our results implied that lung commensal microbiota and host cells might undergo convergent functional alteration and mutually benefit each other.

### Limitations of the study

Our study had several limitations. First, lung tissue samples were cross-sectional and collected from a single center. Second, we did not consider the influence on lung tissue microbiota caused by exposure to cooking fumes, air pollution, or occupational risk factors. Third, as an organ for gas exchange, lung is extensively exposed to environmental microbes. When performing next-generation sequencing of the 16S rRNA gene in low-biomass lung specimens, it would be very difficult to distinguish intrinsic microbial signal from environmental contamination such as microorganisms from the air within the sampling facility and/or people touching the samples. However, we did perform multiple negative controls of DNA extraction blank and no-template amplification in each experimental batch to monitor the potential contamination from laboratory sources such as reagents, glassware, labware, and surfaces. Although involvement of negative controls could exclude contaminant taxa from downstream analysis, it may also remove microbial taxa that are genuinely present in the lung tissue samples in some rare occasion because of technical cross-contamination from tumor samples to control samples. Thus, false positive taxa from environmental contamination might exist while false negative could not be completely ruled out in our results.

## STAR★Methods

### Key resources table

**Table:** 

REAGENT or RESOURCE	SOURCE	IDENTIFIER
**Critical commercial assays**		

Targeted DNA Sequencing Library Preparation kit	Fluidigm	Cat#101-7669
DNeasy Blood & Tissue Kit	Qiagen	Cat#69581
MiSeq Reagent Kit v3, 600 cycles	Illumina	Cat#MS-102-3003

**Deposited data**		

Raw data	This paper	OEP000464

**Software and algorithms**		

RDP Classifier	Cole, J. R. et al. Nucleic Acids Res, 2009	http://rdp.cme.msu.edu/classifier/classifier.jsp
Mothur	Schloss, P. D. et al. Appl Environ Microbiol, 2009	http://www.mothur.org
PICRUSt	Langille, M. G. et al. Nat Biotechnol, 2013	http://picrust.github.com
LEfSe	Segata, N. et al. Genome Biol, 2011	http://huttenhower.sph.harvard.edu/lefse/
R 3.5.3	This paper	http://www.R-project.org/
GATK 3.8	McKenna, A. et al. Genome Res, 2010	https://gatk.broadinstitute.org/

### Resource availability

#### Lead contact

Further information and requests for resources and reagents should be directed to and will be fulfilled by the Lead Contact, Hui Dong (hui.dong@shgh.cn).

#### Materials availability

This study did not generate new unique reagents.

### Experimental model and subject details

All patients involved in this study were enrolled from Shanghai Chest Hospital Affiliated with Shanghai Jiaotong University School of Medicine. The study was approved by the Ethics Committee of Shanghai Chest Hospital Affiliated with Shanghai Jiaotong University School of Medicine (KSY1635) and informed consents were obtained from all subjects.

Tumor and non-tumor lung tissue samples were collected from 158 subjects who underwent thoracoscopic lobectomies. The non-tumor lung tissue samples were resected from an area distant (≥5cm) from the tumor. Tissue samples were snap-frozen in liquid nitrogen within 30 minutes after surgical resection. All the materials and tools used in tissue sampling were sterile. The clinical characteristics of 158 patients were summarized in Table S1. A total of 143 patients were qualified for further analysis after 16S rRNA gene sequencing, including 74 males and 69 females with a mean age of 60.6±10.7 years.

### Method details

#### DNA extraction, 16S rRNA gene amplification and sequencing

DNA was extracted from lung tissue sample following protocols reported by Yu et al. (Yu et al., 2016). Briefly, approximately 30mg tissue sample was homogenized in 1ml lysis buffer (10mM Tris-HCl (pH 8.0), 0.1M EDTA (pH 8.0) and 0.5% SDS) containing proteinase K (0.2 mg/ml) and lysozyme (20mg/ml), and incubated at 56°C with shaking for 2h. The lysate was then applied in DNA extraction using DNeasy Blood & Tissue Kit (Qiagen) according to the manufacturer’s recommendation. The V3-V4 fragments of bacterial 16S rRNA gene were amplified using dual-indexing approach as described previously (Fadrosh et al., 2014). The dual-indexed PCR amplification primers contained not only universal primers targeting V3-V4 hypervariable regions of the 16S rRNA gene, but also a 12bp index and a heterogeneity spacer sequence at both 5’ and 3’ Linker sequences. Since the index and spacer sequences provided more balanced base composition and increased the diversity of sequence reads, the overall sequencing quality of the Illumina MiSeq platform would be greatly improved by applying dual-indexing approach.

The amplified products were purified and then sequenced on MiSeq instrument using the MiSeq® Reagent Kit v3 (600 cycles) (Illumina). After removing raw reads with low quality or without valid index from raw sequencing data, paired sequence reads were assembled. Clean reads were then extracted from assembled sequences by eliminating those had ambiguous bases, those were less than 350 base pairs in length, and those were identified as chimeric sequences. Only clean reads were involved in further analysis. Detailed quality control information for each sequencing run was shown in Table S2.

As all samples were obtained from thoracoscopic lobectomies, they were predicted to be of low microbial biomass. According to the recommendations on dealing with contamination in low microbial biomass microbiome studies (Nejman et al., 2020; Eisenhofer et al., 2019), we took the following precautions to avoid potential contaminations. First, each experimental procedure including DNA extraction, PCR preparation, PCR amplification, PCR product purification, sequencing library preparation and sequencing run was performed in different rooms physically separated and individually ventilated, and filter pipette tips were used for all pipetting. Second, negative controls including DNA extraction blank and no-template amplification controls were involved in each experimental batch and sequenced in each sequencing run. A total of 60 negative controls were successfully sequenced, while human fecal samples were also sequenced in parallel as positive controls to examine the performance of sequencing run. Any operational taxonomic units (OTUs) presented in ≥3 negative controls were regarded as contamination. Contaminated OTUs were then removed from the results of lung tissue samples. After removing contaminated OTUs, we got an average of 51±133 reads per sample remained in negative controls (Table S3), indicating that majority of highly prevalent contaminants have been removed. However, as technical cross-contamination from tumor samples to control samples might occur in some rare occasion, we could not exclude the possibility that removal of contaminated OTUs might lead to elimination of microbial taxa that are genuinely present in the lung tissue samples. Third, in order to minimize batch effect, we randomized tumor and non-tumor tissue samples across five sequencing runs with negative and positive control samples spiked in.

#### Analysis of 16S rRNA gene sequencing data

OTUs were classified based on 97% identity in comparison with SILVA reference database. Taxonomy of each 16S rRNA gene sequence was assigned through the online software RDP classifier (Cole et al., 2009) with a default parameter of confidence threshold of 80%. Indices of alpha diversity, such as Shannon and Simpson index were assessed by Mothur (Schloss et al., 2009). ThetaYC distance was applied in beta diversity analysis. Functional prediction based on the 16S rRNA gene sequence was performed using PICRUSt (Langille et al., 2013). Differences in taxa abundance were analyzed by LEfSe (Segata et al., 2011).

#### Sequencing and analysis of TP53 mutation

Genomic DNA was extracted from tumor and non-tumor lung tissues and submitted for targeted amplification of TP53 gene. The Fluidigm D3 Assay Design program (Fluidigm) was used to design PCR primers for amplicons covering the coding sequence and intron/exon boundaries of TP53. Amplification and preparation of sequencing libraries was performed on LP 192.24 IFC chip using the Targeted DNA Sequencing Library Preparation kit (Fluidigm). Next-generation sequencing was carried out using HiSeq X Ten platform (Illumina). Single nucleotide variants (SNVs) were called from sequencing results by GATK 3.8 package (McKenna et al., 2010), and tumor-specific somatic mutations were derived by filtering out those presented in both tumor and non-tumor samples. Mutations in untranslated regions and introns were excluded as their functional implications are unclear. Furthermore, mutations which were classified as benign or likely benign in ClinVar database (https://www.ncbi.nlm.nih.gov/clinvar/) were regarded as TP53 mutation-negative because such mutations have been proved to be harmless to cell function. Germline mutation was also called, but none was detected in samples involved in this study.

### Quantification and statistical analysis

Statistical analysis was performed in R package (http://www.R-project.org/). Wilcoxon test, paired t test, Fisher’s exact test, Chi-Square test and ANOSIM were applied as appropriate to determine the statistical significance. P < 0.05 was considered statistically significant.

## Data Availability

• Raw 16S rRNA gene sequencing data has been deposited at the National Omics Data Encyclopedia (https://www.biosino.org/node). Accession number is listed in the key resources table. • This paper does not report original code. • Any additional information required to reanalyze the data reported in this paper is available from the lead contact upon request.

## Supplemental information

Document S1. Figures S1–S3

Table S1. Clinical characteristics of patients involved in this study, related to Table 1

Table S2. Quality control information for five sequencing runs on Miseq, related to Figure 1

Table S3. Read counts of 60 negative controls after removing contaminants, related to Figure 1
