# Supplementary material for: Convergent alteration of lung tissue microbiota and tumor cells in lung cancer
Source: iScience. 2021 Dec 16;25(1):103638. doi: 10.1016/j.isci.2021.103638 (PMC8718896; doi:10.1016/j.isci.2021.103638)
Supplement: Document S1. Figures S1–S3 [file mmc1.pdf]

iScience, Volume *25*

Supplemental information

Convergent alteration of lung tissue microbiota and tumor cells in lung cancer

Hui Dong, Qiang Tan, Yuanyuan Xu, Yongqiang Zhu, Yaxian Yao, Yuezhu Wang, Chong Li, Hong Li, Guoqing Zhang, Yan Xiong, Meihua Ruan, Jiadong Zhao, Weirong Jin, Lungen Lu, and Shun Lu

Figure S1. Characteristics of microbiota in tumor and non-tumor lung tissue samples, related to Figure 2.

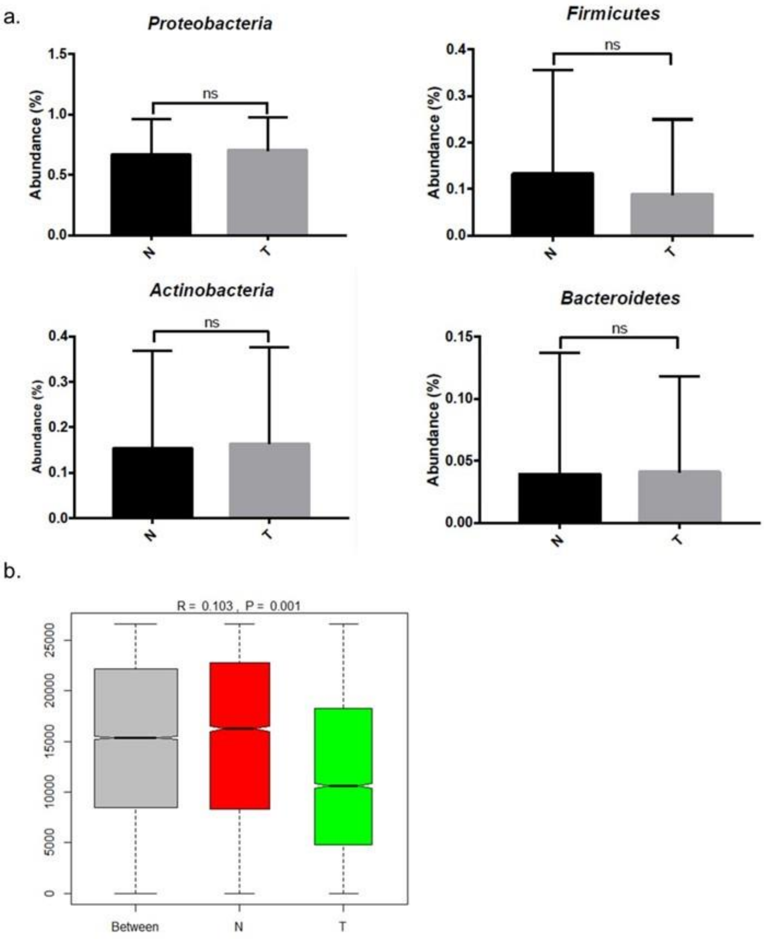

(a). No significant difference was observed in the proportion of *Proteobacteria, Actinobacteria*, *Firmicutes* and *Bacteroidetes* between tumor and non-tumor lung tissue samples. (b). ANOSIM analysis showed significant difference in beta diversity between tumor and non-tumor lung tissue samples (p=0.001). Data are represented as mean ± SD.

Figure S2. Differential microbial taxa in tumor tissue samples of AD and SCC, related to Figure 2.

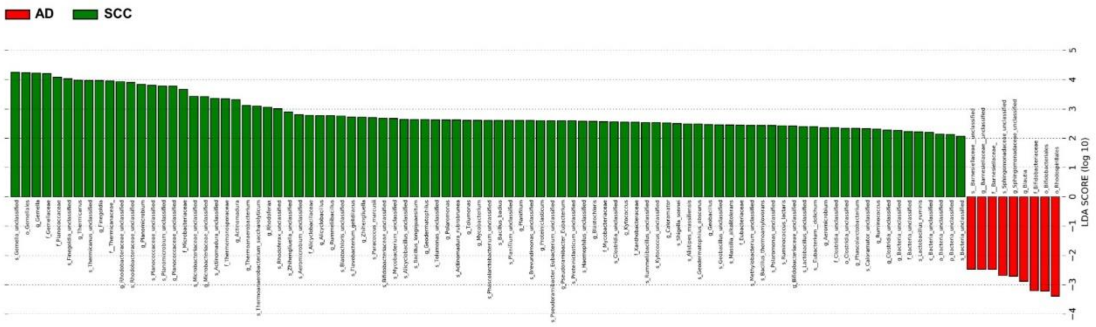

Brevundimonas, Ruminococcus and *Polaromonas* were among the differentially abundant bacterial taxa between AD and SCC.

Figure S3. Differential microbial taxa in TP53 mutation-positive and TP53 mutation-negative tumor tissues, related to Figure 4.

a.

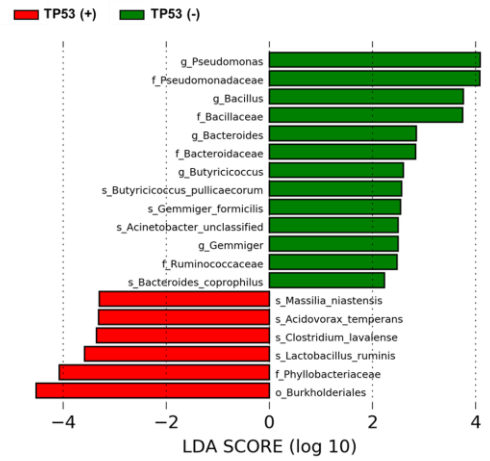

b.

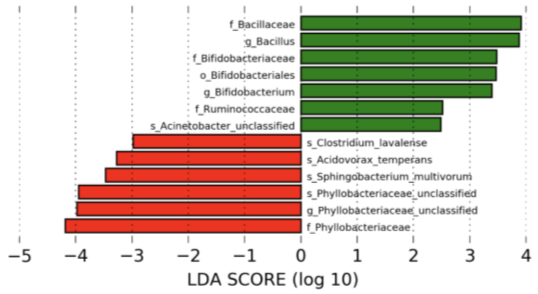

(a). *Acidovorax* and *Massilia* were enriched in TP53(+) tumor tissues compared with TP53(-) tumor tissues regardless of pathological types. (b). *Acidovorax* was enriched in TP53(+) tumor tissues compared with TP53(-) tumor tissues in patients with AD. TP53(+) represents TP53 mutation-positive tumor tissues and TP53(-) represents TP53 mutation-negative tumor tissues.
